# Retraction: Evidence for an African Cluster of Human Head and Body Lice with Variable Colors and Interbreeding of Lice between Continents

**DOI:** 10.1371/journal.pone.0312331

**Published:** 2024-10-14

**Authors:** The PLOS ONE Editors

The *PLOS ONE* Editors retract this article [1, 2] due to concerns about compliance with the PLOS Human Subjects Research policy.

Specifically, the ethics statement of this article [1] states that the study was approved by the Ethics Committee of the Institut Fédératif de Recherche 48 under agreement #12–004. The reported ethics approval document was issued after the study start date. PLOS does not accept retrospective ethics approval. Furthermore, PLOS identified potential competing interests between the approving ethics committee and one or more of the article’s authors.

Additionally, PLOS did not receive information or documentation to verify that the study complied with ethics requirements and research regulations in the countries where samples were collected.

A representative of the Aix-Marseille Université Ethics Committee stated that the institute disagrees with the retraction decision, and that the department’s investigation has not determined that the article violates French regulations or international ethics rules. They also commented that this study focuses exclusively on the analysis of lice and does not involve human subjects, and therefore did not require ethics approval from a Comité de Protection des Personnes according to French law.

All authors either did not respond directly or could not be reached.

## References

[1] Veracx A, Boutellis A, Merhej V, Diatta G, Raoult D (2012) Evidence for an African Cluster of Human Head and Body Lice with Variable Colors and Interbreeding of Lice between Continents. PLoS ONE 7(5): e37804. 10.1371/journal.pone.0037804 22662229 PMC3360600

[2] The *PLOS ONE* Editors (2022) Expression of Concern: Evidence for an African Cluster of Human Head and Body Lice with Variable Colors and Interbreeding of Lice between Continents. PLoS ONE 17(12): e0277634. 10.1371/journal.pone.0277634 36512583 PMC9747002

